# Physical Activity in Centenarians beyond Cut-Point-Based Accelerometer Metrics

**DOI:** 10.3390/ijerph191811384

**Published:** 2022-09-09

**Authors:** Adrián Hernández-Vicente, Jorge Marín-Puyalto, Esther Pueyo, Germán Vicente-Rodríguez, Nuria Garatachea

**Affiliations:** 1 Growth, Exercise, NUtrition and Development (GENUD) Research Group, University of Zaragoza, 50009 Zaragoza, Spain; 2 Department of Physiatry and Nursing, Faculty of Health and Sport Science (FCSD), University of Zaragoza, 22002 Huesca, Spain; 3 Red Española de Investigación en Ejercicio Físico y Salud en Poblaciones Especiales (EXERNET), 50009 Zaragoza, Spain; 4 Biomedical Signal Interpretation and Computational Simulation (BSICoS), Aragón Institute for Engineering Research (I3A), IIS Aragón, University of Zaragoza, 50018 Zaragoza, Spain; 5 CIBER de Bioingeniería, Biomateriales y Nanomedicina (CIBER-BBN), 28029 Madrid, Spain; 6 Centro de Investigación Biomédica en Red de Fisiopatología de la Obesidad y Nutrición (CIBER-Obn), 28029 Madrid, Spain; 7 Instituto Agroalimentario de Aragón, IA2-CITA-Universidad de Zaragoza, 50013 Zaragoza, Spain

**Keywords:** oldest-old, mortality, SPPB, intensity gradient, average acceleration, Mx metrics

## Abstract

This study described and compared physical activity (PA) characteristics at the end of the human lifespan using conventional cut-point-based versus cut-point-free accelerometer metrics. Eighteen institutionalized centenarians (101.5 ± 2.1 years, 72.2% female, 89% frail) wore the wrist GENEActiv accelerometer for 7 days. Conventional metrics, such as time spent in light-intensity PA (LiPA) and moderate-to-vigorous intensity PA (MVPA) were calculated according to published cut-points for adults and older adults. The following cut-point-free metrics were evaluated: average acceleration, intensity gradient and Mx metrics. Depending on the cut-point, centenarians accumulated a median of 15–132 min/day of LiPA and 3–15 min/day of MVPA. The average acceleration was 9.2 mg [Q1: 6.7 mg–Q3: 12.6 mg] and the intensity gradient was −3.19 [−3.34–−3.12]. The distribution of *Z*-values revealed positive skew for MVPA, indicating a potential floor effect, whereas the skew magnitude was attenuated for cut-point-free metrics such as intensity gradient or M5. However, both cut-point-based and cut-point-free metrics were similarly positively associated with functional independence, cognitive and physical capacities. This is the first time that PA has been described in centenarians using cut-point-free metrics. Our results suggest that new analytical approaches could overcome cut-point limitations when studying the oldest-old. Future studies using these new cut-point-free PA metrics are warranted to provide more complete and comparable information across groups and populations.

## 1. Introduction

The population of “oldest-old”, i.e., people aged 80 and over, is growing faster than any other segment of the population and is projected to triple by 2050, reaching 426.4 million worldwide [1]. The increase in total life expectancy is, however, not accompanied by an equivalent increase in healthy life expectancy, with 16–20% of life spent in late-life morbidity [2]. Although aging leads to functional decline of all systems and eventually to death, a physically active lifestyle may attenuate the impact of age on morbidity and mortality [3]. It is well known that physical activity (PA) levels decline with age, with “older adults”, i.e., people aged 65 and over, the most inactive segment of the population [4]. This trend continues to worsen until the end of the human lifespan (>100 years) [5], as maintaining a physically active lifestyle becomes more difficult in the aging population due to various socio-environmental barriers and a progressive lowering of physical functions and capabilities, especially in frail older people [6].

Knowledge-based counseling, including the PA recommendations by the World Health Organization, usually rely on epidemiological associations between objective measures and health outcomes [7]. For this purpose, PA levels should be accurately assessed in all population segments, but very old individuals (i.e., centenarians) remain an understudied population. 

For evaluation of habitual PA, accelerometry is considered as the gold standard since it is an objective method that precisely records bodily accelerations over long periods of time. The analysis of accelerations during the activities of daily living (ADLs) allows researchers to identify the proportion of time spent in sedentary activities or performing PA at different intensities (e.g., light (LiPA), moderate-to-vigorous (MVPA)). PA patterns and relative intensities depend on physiological factors like age or cardiorespiratory fitness. Considering that basal metabolism is substantially lower in older adults compared with the general population, cut-points should be population-specific and protocol-specific [8,9]. Despite recent efforts determining cut-points in people above 70 years of age, there are no established cut-points for 100-year-old or frail individuals. The use of cut-points validated in younger older adults would result in a floor effect when applied to centenarians, with the time spent by centenarians in LiPA or MVPA being under-estimated due to the inappropriateness of the “one size fits all” approach [10].

Novel analytical approaches have been recently developed to assess associations between accelerometer-derived PA measures and health parameters in epidemiological studies. These alternatives include the use of Mx metrics measuring the PA intensity in the X most active minutes [11]. Also, they involve a deeper examination of the intensity distribution throughout the activity profile, calculating the intensity gradient (IG) in combination with a metric of overall PA volume defined by the average acceleration [12]. These cut-point-free measures could help overcome the abovementioned floor effect.

The aim of this study was to describe PA at the end of the human lifespan through cut-point-free accelerometer metrics. The present study also aimed to compare cut-point-based versus cut-point-free accelerometer metrics at two levels: evaluating the floor effect in the different metrics and exploring the associations of the metrics with a positive status in a variety of health outcomes.

## 2. Materials and Methods

### 2.1. Participants

The study population consisted of men and women living in different areas of the region of Aragon in Spain. Only institutionalized individuals reaching at least 100 years of age by the end of the year of the measurements were included. Bedridden centenarians or those going through an acute disease were excluded. Patients with reduced mobility, either helped by their caregivers, using walking stick or walker, and those suffering from chronic diseases or mental disorders such as dementia were included in the study given the high prevalence in the last decades of life. In total, nineteen volunteers (born between 1912 and 1920) were included in the study. After a clear explanation of the potential risks and benefits of the study, all volunteers (or their legally responsible tutor for older adults with cognitive impairments) provided written informed consent to participate in the study. This study was approved by the ethical committee for clinical research of Aragón (ID of the approval: PI18/381). It was conducted by adhering to the Declaration of Helsinki and complying with the European Union General Data Protection Regulation (EU 2016/679).

### 2.2. Protocol

Centenarians were evaluated at their own geriatric nursing home. All the assessments were carried out by the same team of researchers, using the same procedures and equipment. Each participant was evaluated in two sessions. In the first session, volunteers were requested to wear GENEActiv tri-axial accelerometers (ActivInsights Ltd., Cambridgeshire, UK) 24 h/day for 7 consecutive days. The device was mounted on the non-dominant wrist and was set to record accelerations at 10 Hz, which has been demonstrated as sufficient to classify daily activities [13]. GENEActiv accelerometers were initialized and data downloaded in binary format using GENEactiv PC (version 3.2) (ActivInsights Ltd., Cambridgeshire, UK). In the second session, eight days after the first one, accelerometers were collected and health outcomes were assessed in the following order: Health-Related Quality of Life (HRQoL); Mini-Mental State Examination (MMSE); Short Physical Performance Battery (SPPB); Fried’s Frailty Phenotype (FFP); Barthel lndex; and Frailty Trait Scale—short form (FTS-5). 

### 2.3. Health Outcomes

Frailty was assessed by FFP [14] and FTS-5 [15]. FFP classifies a person as frail if 3 or more of the following 5 criteria are met: unintentional weight loss; weak grip strength; self-reported exhaustion; slow walking speed; and low PA. FTS-5 is a shorter version of the Frailty Trait Scale, with similar performance in the diagnosis and evolution of frailty. FTS-5 evaluates 5 domains through 5 items: body mass index, Physical Activity Scale for the Elderly, progressive Romberg test, handgrip strength and walking speed. Each item ranges from 0 (best) to 10 (worst). A total score was calculated as the sum of all item scores (0 to 50) and >25 points was used as the cut-off point to identify frailty [15].

Functional independence was measured using the Spanish version of the Barthel Index of independence during ADLs: feeding, bathing, grooming, dressing, bowel control, bladder control, toileting, chair transfer, ambulation and stair climbing [16]. The index yields a total score out of 100 and allows classification of elders in 5 levels: total dependence (0–20 points), severe dependence (21–60 points), moderate dependence (61–90 points), slight dependence (91–99 points) and independence (100 points) [17]. The sample was dichotomized in 2 groups: “negative outcome” including 6 “totally dependent” centenarians and “positive outcome” including the remaining 13 participants with a score >20 points.

Cognitive capacity was assessed by the Spanish version of the MMSE (from 0–30 points), which is used worldwide to assess global cognitive functioning through the examination of different domains such as orientation to time, orientation to place, registration, attention and calculation, recall, language, repetition and ability to follow commands [18]. The conventional cut-off score for cognitive impairment screening was used to classify subjects according to a dichotomous variable: subjects with ≤23 points were classified in the “negative outcome” group and subjects with 24 points or more were classified in the “positive outcome” group.

Physical capacity was measured using the SPPB test scores (from 1–12 points), depending on performance in: hierarchical standing balance test, gait speed over 4 m and 5-sit-to-stand test [19]. Centenarians were classified in four stages: dependent (1–3 points), frail (4–6 points), pre-frail (7–9 points) and robust (10–12 points) [20]. According to these criteria, 12 centenarians were classified as “dependent” and were included in the “negative outcome” group, whereas the remaining 7 centenarians with a score >3 points were included in the “positive outcome” group.

HRQoL was assessed with the Visual Analog Scale (VAS) of the Spanish EuroQoL-5 Dimension (EQ-5D) questionnaire [21]. The EQ-5D is a standardized HRQoL questionnaire widely used throughout the world. In particular, the VAS is a vertical scale ranging from 0 “worst imaginable health state” to 100 “best imaginable health state” and participants are asked to tick the level they think their current health corresponds to. VAS cut-off values were extracted from the available normative data (Spanish values), corresponding to 66.7 for older males (≥75 y) and 59.4 in the case of older females (≥75 y) [22]. Participants who had a VAS score below their cut-off value were classified in the “negative outcome” group, whereas participants who showed a HRQoL above the cut-off value were classified in the “positive outcome” group.

Date of birth and date of death were obtained from the Spanish National Dead Index (Ministry of Health, Consumer Affairs and Social Welfare). The study population was followed up for 1.5 years from baseline. Early mortality was defined as all-cause mortality within 1 year following the measurements. Centenarians in the early mortality group (≤1 year) were classified as “negative outcome” and those in the survival group were classified as “positive outcome”.

### 2.4. Accelerometer Processing

The analysis of accelerometry data was carried out using the GGIR 2.3-0 [23] package of the statistical programming language R v.3.5.1. Non-wear time detection and minimum valid time requirements for each accelerometry register were evaluated using GGIR’s default settings to facilitate comparability with previous studies. The minimum valid hours per day were set at 16, whereas the minimum valid days per record was established as 3, irrespective of whether they were weekdays or weekends, considering that this population does not follow a labor-related calendar. Table 1 includes the relationship between the nomenclature used throughout this paper and the variable names from GGIR output.

Time spent in LiPA (18–60 mg) and MVPA (>60 mg) were calculated using the sensitivity optimized cut-points proposed by Migueles et al. [8]. These cut-points were established based on a population of older adults (≥70 years old) but not the oldest elders. Moreover, data were also analyzed using other previously reported cut-points based on Euclidean Norm Minus One *G* (ENMO), such as the cut-points for adults published by Hildebrand et al., (LiPA: 45.8–93.2 mg; MVPA: >93.2 mg) [24,25] and the cut-points for older adults published by Sanders et al., (LiPA: 57–104 mg; MVPA: >104 mg) [26].

In addition to these traditionally used metrics, recently proposed approaches were evaluated [27], including the average acceleration, the IG and the Mx metrics [12]. Average acceleration [12] reflects the average acceleration throughout the entire measurement period and can be used as a proxy for total daily PA-related energy expenditure [27] or PA volume. IG, calculated as the slope (negative) of the linear regression between natural logs of time and acceleration intensity, captures the distribution of PA intensity across all levels.

Mx metrics [11] evaluate the most active X minutes from a participant’s daily activity (e.g., M30 refers to the acceleration above which the most active 30 min were spent), which can in turn be used to describe the distribution of intensities across different time frames and to establish a direct comparison with health-related PA guidelines. Here, the intensity levels corresponding to the most active 1, 5, 15, 30, 60, 120 and 480 min were recorded.

### 2.5. Statistical Analysis

Statistical analyses were carried out using the statistical software Jamovi v.2.2.5. (The jamovi project, https://www.jamovi.org, accessed on 7 September 2022). The statistical significance was set at an alpha value of 0.05. Descriptive values for all previously defined variables were obtained and the skewness and kurtosis of the variable distributions were quantified to detect a potential floor effect. Standardized values were calculated for each variable to allow comparability among them.

Given that the normality assumption was violated, as checked with the Shapiro–Wilk normality test, non-parametric tests were selected. After dividing the sample into dichotomous groups according to the abovementioned health and functional outcomes, Mann–Whitney U tests and their associated effect sizes were used to identify differences in LiPA, MVPA, average acceleration, IG and Mx metrics between groups.

## 3. Results

### 3.1. Participants

Eighteen out of nineteen centenarians had accelerometry registers that met the inclusion criteria for the analysis. Only one subject was excluded due to issues with the accelerometer during the recording. Table 2 shows the descriptive characteristics of the centenarians: 72.2% of them were women and almost all were frail, i.e., 83.3% according to FFP and 88.9% according to FTS-5 [14,15].

### 3.2. Descriptive Accelerometry Results

Table 3 shows the descriptive results obtained by accelerometry. Depending on the cut-point, centenarians accumulated a median of 132 min/day [Interquartile range (IQR): 129.5 min/day] to 14.6 min/day [IQR: 32 min/day] of LiPA and 15.5 min/day [IQR: 36.7 min/day] to 3.3 min/day [IQR: 7.9 min/day] of MVPA. The cut-point-free measures for average acceleration (proxy for PA volume) and IG (proxy for PA intensity) were 9.2 mg [IQR: 5.9 mg] and −3.19 [IQR: 0.22], respectively.

### 3.3. Comparison of Cut-Point-Based and Cut-Point-Free Approaches

**Figure 1:**
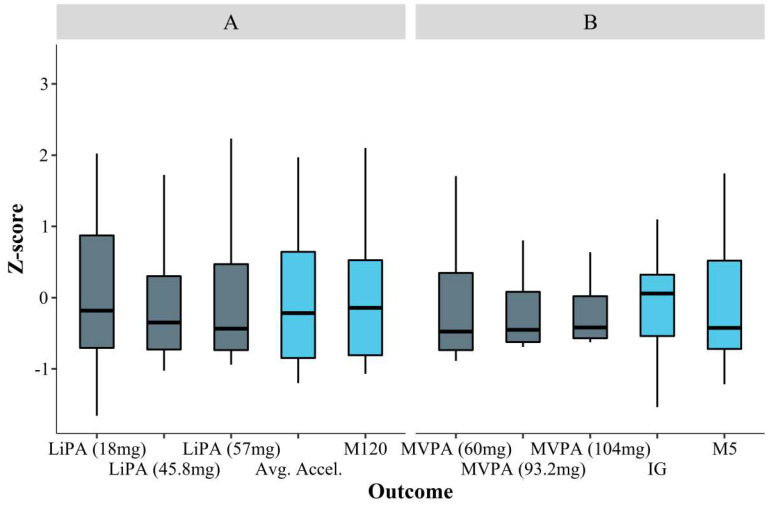
Box plots representing the distribution of *Z*-values for the conventional (grey) and cut-point-free (blue) physical activity accelerometer metrics, corresponding to: (**A**) physical activity volume and (**B**) intensity. LiPA = Light-intensity physical activity; Avg.Accel. = Average acceleration; M120 = Acceleration above which the most active 120 min of the day are accumulated. MVPA = Moderate-to-vigorous physical activity; IG = Intensity gradient; M5 = Acceleration above which the most active 5 min of the day are accumulated.

Figure 1 shows box plots representing the distribution of the *Z*-values for the conventional and cut-point-free PA accelerometer metrics. Regarding PA volume variables (see Figure 1A), no remarkable differences were observed in the floor effect between cut-point-based (LiPA) and cut-point-free metrics (i.e., Avg. Accel. and M120). Table 3 shows a positive skew for MVPA (Skewness 1.13 to 2.52), indicating a potential floor effect for the conventional cut-points as hypothesized. The magnitude of the skew and kurtosis was attenuated for the cut-point-free metrics. Similar observations can be made from Figure 1B, presenting PA intensity variables.

Differences between dichotomous groups of health outcomes according to conventional and cut-point-free PA accelerometer metrics are presented in Table 4. Both cut-point-based and cut-point-free metrics presented significant associations with variables such as the Barthel Index, MMSE or SPPB. Only the cut-point-free index M30 was significantly associated with 1-year survival.

Descriptive results for the dichotomized groups presenting significant differences are displayed in Table 5. Centenarians who survived more than 1-year presented a median of 36.8 mg [IQR: 19.8 mg] for M30, whereas those who died in the year following the measurements reported a median of 19.6 mg [IQR: 7.7 mg] for M30.

## 4. Discussion

The daily time spent by a centenarian in LiPA and MVPA varies greatly depending on the cut-point used for the calculation. Our centenarians showed a potential floor effect in MVPA, being intensified in the most exigent cut-points. Regarding cut-point-free metrics, decreased PA volume and intensity could be observed in centenarians compared with values reported in younger populations. This age-related decrease in PA volume and intensity up to the limit of human lifespan should be confirmed in future studies providing more extensive characterization in the 80–100-year-old population. The advantages and disadvantages of cut-point-free metrics with respect to cut-point-based metrics are discussed below.

### 4.1. Descriptive Accelerometry Results

To the best of our knowledge, there is only one published study that assessed PA levels in centenarians using accelerometry [5]. That study evaluated conventional metrics and, thus, just a few of the reported results can be directly compared. In addition, it should be taken into account that the cut-points, brand and location of the accelerometer were different between studies. The centenarians in [5] accumulated a mean of 63 min/day of active time (LiPA and MVPA), with the centenarians in the present study accumulating 17.9 to 147.5 min/day depending on the cut-point used. One of the main conclusions from [5] was that the decline in PA levels continues to worsen until the end of the human lifespan [5], which should be interpreted with caution, as it depends on the cut-point used in the evaluation. As an example, if the lowest cut-point reported in [8] were used, our centenarians would perform a median of 147.5 min/day of active time, this being higher than the 98 min/day reported in nonagenarians [5], or the 117.6 min/day measured in subjects aged 85 years or older [28]. 

The selection of a cut-point is avoided when using cut-point-free metrics, since they are not based on intensity thresholds. There are currently no available cut-point-free data for octogenarians and nonagenarians. In this study, average acceleration in centenarians was 9.2 mg. Previous studies have reported values of 27.1 mg and 34.3 mg in populations of postmenopausal women and 13–14 year adolescent girls, respectively [29]. Considering that more than 7.2% of all-cause deaths and up to 8% of non-communicable diseases are attributable to physical inactivity [30], it is worrying that the most rapidly growing subgroup of the population (i.e., oldest-old) is highly inactive. The physical inactivity of the oldest-old should be viewed as an urgent priority for policy makers, given its implications for HRQoL and the associated healthcare cost [31].

For another cut-point-free metric like IG, a decline with age has been reported: −1.96 (sample mean age: 9.6 years), −2.19 (12.3 years), −2.28 (13.6 years), −2.55 (41.2 years), −2.66 (46.2 years), −2.74 (59.0 years), −2.74 (64.2 years) [29]. In concordance with this, our centenarians rendered an IG value of −3.19 [IQR: 0.22]. These results can be interpreted in light of the independent positive association between IG and physical function [12] and the fact that aging leads to the functional decline of all systems [3].

**Figure 2:**
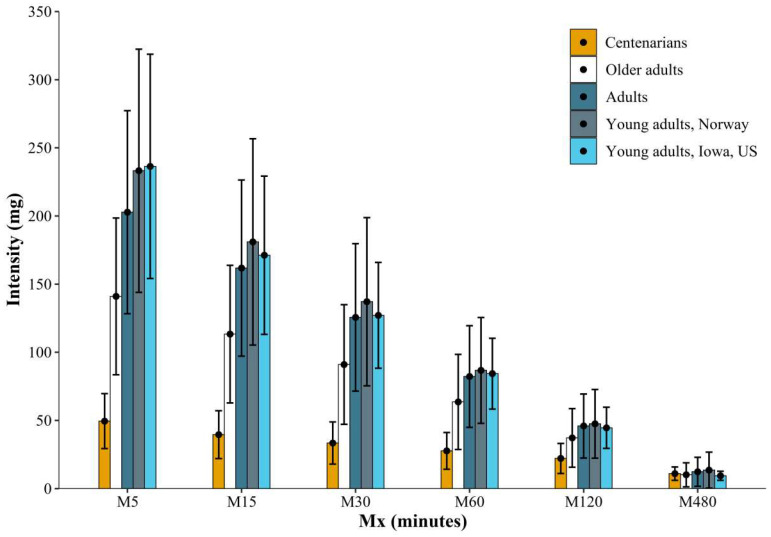
Mx metrics during the lifespan. Mx metrics = acceleration above which a person’s most active X minutes are accumulated (i.e., M120 refers to the intensity at which the most active 120 min of the day were spent). Young adults, Adults and Older adults are normative data from Norway by HS Rosfjord in the University of Adger: “New metrics for analysis and presentation of device-based indices of physical activity” [35]; and Young adults from US are data from Rowlands et al. [36]. Note that physical activity in [35,36] was measured using ActiGraph GT3X+ accelerometers worn at the hip.

Regarding Mx metrics, an age-related decrease can be observed for all Mx durations, being more pronounced for short-duration Mx, see Figure 2. According to these results, centenarians perform all their efforts at a more similar intensity, whereas young people can reach high intensities in short-duration efforts. Nevertheless, our results should be interpreted with caution, since our sample is only composed of institutionalized centenarians. A broad variation in functional capacity can be observed in centenarian populations, with some centenarians performing all ADLs and others being bedridden [32], resulting in a wide range (2–89%, mean 37.3%) of centenarians living in geriatric nursing homes across European countries [33]. Due to the aforementioned selection bias, certain centenarian populations could show equal or even improved results than those of older adults. In fact, there are some inspirational examples of centenarian athletes who continue participating in sport competitions, including marathon or 1500 m swimming [34].

### 4.2. Comparison of Cut-Point-Based and Cut-Point-Free Approaches

The limitations of cut-points are well known and lead to some problems that we have noticed throughout this article [37]. First, cut-points are protocol- (e.g., accelerometer placement) and population- (e.g., age group) specific, therefore: (i) results are not comparable across studies; (ii) the time spent by a centenarian in LiPA and MVPA can vary greatly depending on the cut-point used; and (iii) scientists have to select one among the many available cut-points for a population e.g., older adults, with no cut-points available for specific age segments such as centenarians [8,26]. Cut-point-free metrics emerged as a solid alternative since they are population independent, although are wear-site specific and may differ between some brands of monitors [11].

A second limitation of cut-points is that two participants score very different if one has activity falling just above the cut-point and one has activity falling just below the cut-point [37]. These crude boundaries between intensity levels do not exist in human physiology. Third and last, many participants fail to reach any activity above cut-points (particularly in the vigorous range) [37]. In particular, our centenarians showed a potential floor effect in MVPA, being intensified in the most exigent cut-points. This is avoided when using cut-point-free metrics, with no participant scoring near zero values. We expected a more attenuated right-skewed distribution for the variables that cover the intensity spectrum continuously (i.e., average acceleration and IG) than for Mx metrics, which was confirmed for average acceleration while IG skewness was similar to that of the Mx metrics [11].

With the Mx approach, data are not collapsed into categories but the continuous nature of the data is maintained and post hoc interpretations can be made in relation to any cut-point (e.g., in order to see the prevalence of meeting PA guidelines) and/or accelerations indicative of typical activities, facilitating the development of public-health-friendly recommendations [11]. Moreover it should be highlighted that in the Mx metrics, minutes can be accumulated in any way across the day, with no need for the activity to be in bouts, being coherent with the “*every move counts*” perspective from the World Health Organization PA recommendations [7]. As an example, Rowlands et al. [37] estimated MVPA thresholds representative of a brisk walk (170 mg) or a fast walk (250 mg) for adults, and our centenarians do not reach those accelerations even in M_1_. However, the descriptive data presented in this manuscript can be compared in the future with as many alternative cut-points as needed, for example if the VO_2net_ age-equivalent cut-points were expanded to the whole human lifespan [38]. In the same vein, research in the area of cut-point-based metrics is moving towards post-data collection approaches such as personalized accelerometer cut-points using machine learning [10].

Previous studies stated that: “Future research should assess how the PA profile is related with health outcomes by age and disease categories with a view to informing accelerometer-driven PA prescriptions and recommendations” [39]. The present study responds to this demand, providing an insight into the PA profile of people who have lived 20–30 years longer than the average Westerner. The dose–response associations between PA and health are so strong [40] that, despite its limitations, conventional cut-point-based metrics also found differences between groups with “negative” vs. “positive” functional independence, cognitive capacity and physical capacity. Therefore, conventional cut-point-based metrics preserve a certain clinical utility since they are capable of identifying health status in our sample.

Currently the World Health Organization PA guidelines recommend that older adults should be as physically active as their functional ability allows [7]. The present study provides PA profile descriptive results for a specific population, i.e., institutionalized centenarians, as well as for subgroups associated with “positive outcome” in different health variables, see Table 5. As an example, if the proposed 70 mg threshold representative of a slow walking for adults is applied [36], descriptive results show that those centenarians with “positive outcome” in functional independence, cognitive capacity and physical capacity, were able to accumulate 1 min per day (i.e., M1) at the intensity of “slow walking”. This information may result in evidence-based PA guidelines for institutionalized centenarians or as an objective for maintaining these specific health outcomes until the end of our lives. Moreover, the applicability of accelerometers is not restricted to evaluation of PA outcomes. When accelerometer-driven PA guidelines are available for the oldest old, accelerometers could be used to motivate them to reach evidence-based goals on PA intensity, duration, timing or type [12].

### 4.3. Strengths and Limitations

The present study has several main strengths. One of them is the exceptionality of the sample, particularly considering that being a centenarian is a rare phenotype, 21.6/100,000 of Europe inhabitants [41]. The percentage of women in our sample was similar to the overall centenarian population in Europe (83% women) [33]. This study is novel and represents the first study in centenarians that assessed PA using cut-point-free metrics. Another important strength of our study is the measurement of PA in everyday life during a whole week using a validated device and calculating different cut-point-based variables. In addition, several health outcomes were measured, with centenarians being followed up after a year for early mortality. Therefore, the study is not merely descriptive, but also explores the relationship between the PA profile and health outcomes. Last but not least, the study makes a comparison of cut-point-based and cut-point-free approaches and discusses the possible future application of accelerometers in centenarian populations.

On the other hand, the main limitation to be acknowledged is the specificity of the sample. All the participants are Spanish, white and institutionalized. Consequently, generalization of our results could be partly limited. In future research, samples including non-institutionalized centenarians would allow to complete the image, expanding and confirming the results obtained by this study.

## 5. Conclusions

This is the first time that PA has been described in centenarians using cut-point-free metrics. In line with literature reports describing that cut-point-free metrics present an age-related reduction in PA volume and intensity, our centenarians had the lowest values in all the variables. This is in contrast to cut-point-based metrics such as MVPA that presented a floor effect, suggesting that cut-point-free approaches could overcome cut-point-based metric limitations when studying the oldest-old. Both cut-point-based and cut-point-free measures were related to health states, but the cut-point-free M30 was the only one related to early mortality. Future studies are warranted to confirm the value of the cut-point-free PA metrics in centenarians.

## Figures and Tables

**Table 1:** Correspondence between the nomenclature of the present paper and variable names from GGIR outputs.

Nomenclature in This Paper	Variable Names in GGIR	Results File in GGIR
LiPA	dur_day_total_LIG_min_pla	Part 5
MVPA	dur_day_total_MOD_min_pla	Part 5
Average acceleration	AD_mean_ENMO_mg_0-24hr	Part 2
Intensity gradient	AD_ig_gradient_ENMO_0-24hr	Part 2
M1	p99.93_ENMO_mg_0-24hr_fullRecording	Part 2
M5	p99.65_ENMO_mg_0-24hr_fullRecording	Part 2
M15	p98.96_ENMO_mg_0-24hr_fullRecording	Part 2
M30	p97.92_ENMO_mg_0-24hr_fullRecording	Part 2
M60	p95.83_ENMO_mg_0-24hr_fullRecording	Part 2
M120	p91.67_ENMO_mg_0-24hr_fullRecording	Part 2
M480	p66.67_ENMO_mg_0-24hr_fullRecording	Part 2

**Table 2:** Outcome measures (mean and SD) for the overall group and for each dichotomized group according to health outcomes.

Outcome	Overall (*N* = 18)		Negative Outcome			Positive Outcome		
	Mean	SD	*N*	Mean	SD	*N*	Mean	SD
Age (years)	101.5	2.1	-	-	-	-	-	-
FFP (5–0)	3.3	0.9	-	-	-	-	-	-
FTS-5 (50–0)	33.1	6.1	-	-	-	-	-	-
Barthel (0–100)	39.7	23.5	6	17.5	2.7	12	50.8	21.2
MMSE (0–30)	22.1	5.9	9	17.6	5.1	9	26.6	1.4
SPPB (0–12)	2.9	2.8	11	1.3	1.3	7	5.6	2.4
VAS (0–100)	60.3	33.5	7	24.3	19.0	11	83.2	14.5
1-year survival	-	-	6	-	-	12	-	-

The scoring ranges for the outcomes are expressed as (worst score—best score). FFP = Fried’s Frailty Phenotype; FTS-5 = Frailty Trait Scale—short form; Barthel = Barthel Index of independence during activities of daily living; MMSE = Mini-Mental State Examination; SPPB = Short Physical Performance Battery; VAS = Visual Analog Scale of health-related quality of life. For each health outcome the sample was dichotomized into “negative outcome” and “positive outcome” groups. SD = Standard deviation.

**Table 3:** Descriptive results of physical activity in centenarians.

Outcome	Minimum	Q1	Median	Q3	Maximum	Skewness	Kurtosis
LiPA 18 mg (min/day)	11.2	89.5	132.0	219.0	313.0	0.321	−0.735
LiPA 45.8 mg (min/day)	1.8	13.1	27.4	52.1	118.0	1.080	0.032
LiPA 57 mg (min/day)	1.2	6.6	14.6	38.6	85.3	1.180	0.279
MVPA 60 mg (min/day)	1.6	6.7	15.5	43.4	105.0	1.130	−0.135
MVPA 93.2 mg (min/day)	0.6	1.7	4.4	13.1	60.8	2.100	4.370
MVPA 104 mg (min/day)	0.4	1.3	3.3	9.2	53.5	2.520	6.900
Avg. Accel. (mg)	5.3	6.7	9.2	12.6	17.9	0.696	−0.786
IG	−3.59	−3.34	−3.19	−3.12	−2.46	1.260	3.410
Mx metrics (mg)							
M480	5.7	7.1	9.6	13.3	21.1	0.989	−0.038
M120	10.3	13.2	20.5	27.9	45.2	0.758	−0.566
M60	13.3	16.3	24.8	34.7	57.3	0.792	−0.372
M30	15.8	20.0	29.0	40.9	68.4	0.858	−0.142
M15	18.8	25.1	33.6	47.9	80.2	0.949	0.193
M5	25.0	35.0	40.9	59.9	97.7	1.010	0.476
M1	33.0	46.4	58.8	78.8	132.0	1.270	2.090

LiPA = Light-intensity physical activity; MVPA = Moderate-to-vigorous physical activity; Avg.Accel. = Average acceleration; IG = Intensity gradient; Mx metrics = Acceleration above which a person’s most active X minutes (Mx) are accumulated.

**Table 4:** Differences in physical activity variables between dichotomous groups for different health outcomes.

	PA Variable	Barthel		MMSE		SPPB		VAS		1-Year Survival	
Outcome		U	ES	U	ES	U	ES	U	ES	U	ES
LiPA 18 mg		13 *	0.639	7 *	0.827	12 *	0.688	34	0.117	18	0.500
LiPA 45.8 mg		7 *	0.806	9 *	0.778	6 *	0.844	36	0.065	19	0.472
LiPA 57 mg		7 *	0.806	11 *	0.728	5 *	0.870	35	0.091	20	0.444
MVPA 60 mg		7 *	0.806	14 *	0.654	5 *	0.870	34	0.117	19	0.472
MVPA 93.2 mg		6 *	0.833	15 *	0.630	5 *	0.870	32	0.169	20	0.444
MVPA 104 mg		4 *	0.889	15 *	0.630	5 *	0.870	30	0.221	22	0.389
Avg. Accel.		9 *	0.750	13 *	0.679	7 *	0.818	33	0.143	15	0.583
IG		9 *	0.750	15 *	0.630	11 *	0.714	31	0.195	26	0.278
Mx metrics											
M480		10 *	0.722	11 *	0.728	7 *	0.818	33	0.143	17	0.528
M120		8 *	0.778	14 *	0.654	5 *	0.870	33	0.143	16	0.556
M60		9 *	0.750	14 *	0.654	5 *	0.870	34	0.117	15	0.583
M30		10 *	0.722	15 *	0.630	6 *	0.844	33	0.143	14*	0.611
M15		11 *	0.694	17 *	0.580	7 *	0.818	34	0.117	16	0.556
M5		9 *	0.750	17 *	0.580	5 *	0.870	34	0.117	18	0.500
M1		10 *	0.722	16 *	0.605	6 *	0.844	35	0.091	21	0.417

PA = Physical activity; LiPA = Light-intensity physical activity; MVPA = Moderate-to-vigorous physical activity; Avg. Accel. = Average acceleration; IG = Intensity gradient; Mx metrics = Acceleration above which a person’s most active X minutes (Mx) are accumulated. * = Significant differences between “negative outcome” and “positive outcome” groups (*p* ≤ 0.05, Mann–Whitney U test). U = U statistic. ES = Effect size. Barthel = Barthel Index of independence during activities of daily living; MMSE = Mini-Mental State Examination; SPPB = Short Physical Performance Battery; VAS = Visual Analog Scale of health-related quality of life.

**Table 5:** Descriptive results of physical activity in the dichotomized groups of centenarians (median and interquartile range) for the health outcomes with statistical differences.

	PA Variable	Barthel		MMSE		SPPB	
Outcome		Negative (*N* = 6)	Positive (*N* = 12)	Negative (*N* = 9)	Positive (*N* = 9)	Negative (*N* = 11)	Positive (*N* = 7)
LiPA 18 mg		81.5 (53.8)	169 (115)	89.1 (40.6)	209 (85.0)	92.8 (68.9)	231 (83.0)
LiPA 45.8 mg		10.3 (8.0)	51.0 (54.2)	14.0 (11.2)	52.3 (73.1)	14.0 (19.5)	65.3 (58.6)
LiPA 57 mg		5.4 (3.8)	32.1 (36.5)	7.1 (7.6)	34.5 (53.5)	7.1 (10.4)	41.3 (37.2)
MVPA 60 mg		5.7 (3.5)	35.0 (65.8)	6.9 (8.1)	39.0 (61.9)	6.9 (10.7)	79.0 (46.9)
MVPA 93.2 mg		1.5 (0.8)	10.4 (19.7)	1.7 (2.8)	12.5 (19.4)	1.7 (2.9)	23.8 (21.3)
MVPA 104 mg		1.1 (0.5)	7.2 (13.4)	1.4 (1.9)	8.7 (13.0)	1.4 (2.2)	16.3 (15.8)
Avg. Accel.		6.7 (1.4)	11.2 (6.8)	6.5 (1.5)	10.5 (5.3)	7.2 (3.0)	15.1 (4.6)
IG		−3.44 (0.18)	−3.15 (0.13)	−3.35 (0.19)	−3.14 (0.08)	−3.29 (0.27)	−3.10 (0.19)
Mx metrics							
M480		7.3 (1.8)	12.2 (6.5)	6.8 (1.9)	11.1 (7.8)	7.9 (3.3)	14.6 (7.5)
M120		12.1 (3.3)	24.8 (17.7)	13.2 (4.4)	25.9 (13.3)	13.2 (9.2)	35.3 (11.9)
M60		15.8 (3.8)	32.3 (20.5)	16.4 (4.3)	33.8 (13.1)	16.4 (9.7)	42.4 (13.5)
M30		20.2 (4.6)	39.2 (22.9)	20.8 (4.2)	40.7 (13.4)	20.8 (9.5)	49.5 (15.6)
M15		24.7 (6.0)	45.6 (24.2)	25.6 (6.5)	46.0 (12.3)	25.6 (9.8)	55.4 (18.2)
M5		33.8 (6.7)	57.2 (26.9)	35.8 (6.1)	59.0 (14.8)	35.8 (9.3)	67.0 (18.3)
M1		44.8 (4.0)	70.5 (26.0)	46.8 (10.4)	74.9 (15.3)	46.8 (16.0)	81.4 (17.2)

PA = Physical activity; LiPA = Light-intensity physical activity; MVPA = Moderate-to-vigorous physical activity; Avg. Accel. = Average acceleration; IG = Intensity gradient; Mx metrics = Acceleration above which a person’s most active X minutes (Mx) are accumulated. For each health outcome the sample was dichotomized into negative = “negative outcome” and positive = “positive outcome” groups. Barthel = Barthel index of independence during activities of daily living; MMSE = Mini-Mental State Examination; SPPB = Short Physical Performance Battery.

## Data Availability

The datasets analyzed during the current study are available from the corresponding author on reasonable request.

## References

[1] United Nations, Department of Economic and Social Affairs Population Division (2020). World Population Ageing 2019.

[2] Partridge L., Deelen J., Slagboom P.E. (2018). Facing up to the Global Challenges of Ageing. Nature.

[3] Harridge S.D.R., Lazarus N.R. (2017). Physical Activity, Aging, and Physiological Function. Physiology.

[4] Troiano R.P., Berrigan D., Dodd K.W., Mâsse L.C., Tilert T., Mcdowell M. (2008). Physical Activity in the United States Measured by Accelerometer. Med. Sci. Sports Exerc..

[5] Hernández-Vicente A., Santos-Lozano A., Mayolas-Pi C., Rodríguez-Romo G., Pareja-Galeano H., Bustamante N., Gómez-Trullén E.M., Lucia A., Garatachea N. (2019). Physical Activity and Sedentary Behavior at the End of the Human Lifespan. J. Aging Phys. Act..

[6] Franco M.R., Tong A., Howard K., Sherrington C., Ferreira P.H., Pinto R.Z., Ferreira M.L. (2015). Older People’s Perspectives on Participation in Physical Activity: A Systematic Review and Thematic Synthesis of Qualitative Literature. Br. J. Sports Med..

[7] Bull F.C., Al-Ansari S.S., Biddle S., Borodulin K., Buman M.P., Cardon G., Carty C., Chaput J.P., Chastin S., Chou R. (2020). World Health Organization 2020 Guidelines on Physical Activity and Sedentary Behaviour. Br. J. Sports Med..

[8] Migueles J.H., Cadenas-Sanchez C., Alcantara J.M.A., Leal-Martín J., Mañas A., Ara I., Glynn N.W., Shiroma E.J. (2021). Calibration and Cross-Validation of Accelerometer Cut-Points to Classify Sedentary Time and Physical Activity from Hip and Non-Dominant and Dominant Wrists in Older Adults. Sensors.

[9] Leal-Martín J., Muñoz-Muñoz M., Keadle S.K., Amaro-Gahete F., Alegre L.M., Mañas A., Ara I. (2021). Resting Oxygen Uptake Value of 1 Metabolic Equivalent of Task in Older Adults: A Systematic Review and Descriptive Analysis. Sport. Med..

[10] Nnamoko N., Cabrera-Diego L.A., Campbell D., Sanders G., Fairclough S.J., Korkontzelos I. (2021). Personalised Accelerometer Cut-Point Prediction for Older Adults’ Movement Behaviours Using a Machine Learning Approach. Comput. Methods Programs Biomed..

[11] Rowlands A.V., Dawkins N.P., Maylor B., Edwardson C.L., Fairclough S.J., Davies M.J., Harrington D.M., Khunti K., Yates T. (2019). Enhancing the Value of Accelerometer-Assessed Physical Activity: Meaningful Visual Comparisons of Data-Driven Translational Accelerometer Metrics. Sport. Med. Open.

[12] Rowlands A.V., Edwardson C.L., Davies M.J., Khunti K., Harrington D.M., Yates T. (2018). Beyond Cut Points: Accelerometer Metrics That Capture the Physical Activity Profile. Med. Sci. Sports Exerc..

[13] Zhang S., Murray P., Zillmer R., Eston R.G., Catt M., Rowlands A.V. (2012). Activity Classification Using the Genea: Optimum Sampling Frequency and Number of Axes. Med. Sci. Sports Exerc..

[14] Fried L.P., Tangen C.M., Walston J., Newman A.B., Hirsch C., Gottdiener J., Seeman T., Tracy R., Kop W.J., Burke G. (2001). Frailty in Older Adults: Evidence for a Phenotype. J. Gerontol. Ser. A Biol. Sci. Med. Sci..

[15] García-García F.J., Carnicero J.A., Losa-Reyna J., Alfaro-Acha A., Castillo-Gallego C., Rosado-Artalejo C., Gutiérrrez-Ávila G., Rodriguez-Mañas L. (2020). Frailty Trait Scale–Short Form: A Frailty Instrument for Clinical Practice. J. Am. Med. Dir. Assoc..

[16] Cabañero-Martínez M.J., Cabrero-García J., Richart-Martínez M., Muñoz-Mendoza C.L. (2009). The Spanish Versions of the Barthel Index (BI) and the Katz Index (KI) of Activities of Daily Living (ADL): A Structured Review. Arch. Gerontol. Geriatr..

[17] Shah S., Vanclay F., Cooper B. (1989). Improving the Sensitivity of the Barthel Index for Stroke Rehabilitation. J. Clin. Epidemiol..

[18] Lobo A., Saz P., Marcos G., Día J.L., de la Cámara C., Ventura T., Morales Asín F., Fernando Pascual L., Montañés J.A., Aznar S. (1999). Revalidation and Standardization of the Cognition Mini-Exam (First Spanish Version of the Mini-Mental Status Examination) in the General Geriatric Population. Med. Clin..

[19] Guralnik J.M., Simonsick E.M., Ferrucci L., Glynn R.J., Berkman L.F., Blazer D.G., Scherr P.A., Wallace R.B. (1994). A Short Physical Performance Battery Assessing Lower Extremity Function: Association with Self-Reported Disability and Prediction of Mortality and Nursing Home Admission. J. Gerontol..

[20] Izquierdo M. (2019). Multicomponent Physical Exercise Program: Vivifrail. Nutr. Hosp..

[21] Badia X., Roset M., Montserrat S., Herdman M., Segura A. (1999). The Spanish Version of the EuroQol: Description and Applications. Med. Clin..

[22] Szende A., Janssen B., Cabasés J. (2014). Self-Reported Population Health: An International Perspective Based on EQ-5D.

[23] Migueles J.H., Rowlands A.V., Huber F., Sabia S., van Hees V.T. (2019). GGIR: A Research Community–Driven Open Source R Package for Generating Physical Activity and Sleep Outcomes from Multi-Day Raw Accelerometer Data. J. Meas. Phys. Behav..

[24] Hildebrand M., Hansen B.H., van Hees V.T., Ekelund U. (2017). Evaluation of Raw Acceleration Sedentary Thresholds in Children and Adults. Scand. J. Med. Sci. Sport..

[25] Hildebrand M., Van Hees V.T., Hansen B.H., Ekelund U. (2014). Age Group Comparability of Raw Accelerometer Output from Wrist-and Hip-Worn Monitors. Med. Sci. Sports Exerc..

[26] Sanders G.J., Boddy L.M., Sparks S.A., Curry W.B., Roe B., Kaehne A., Fairclough S.J. (2019). Evaluation of Wrist and Hip Sedentary Behaviour and Moderate-to-Vigorous Physical Activity Raw Acceleration Cutpoints in Older Adults. J. Sports Sci..

[27] Migueles J.H., Aadland E., Andersen L.B., Brønd J.C., Chastin S.F., Hansen B.H., Konstabel K., Kvalheim O.M., McGregor D.E., Rowlands A.V. (2021). GRANADA Consensus on Analytical Approaches to Assess Associations with Accelerometer-Determined Physical Behaviours (Physical Activity, Sedentary Behaviour and Sleep) in Epidemiological Studies. Br. J. Sports Med..

[28] Davis M.G., Fox K.R., Hillsdon M., Sharp D.J., Coulson J.C., Thompson J.L. (2011). Objectively Measured Physical Activity in a Diverse Sample of Older Urban UK Adults. Med. Sci. Sports Exerc..

[29] Rowlands A.V., Fairclough S.J., Yates T., Edwardson C.L., Davies M., Munir F., Khunti K., Stiles V.H. (2019). Activity Intensity, Volume, and Norms: Utility and Interpretation of Accelerometer Metrics. Med. Sci. Sports Exerc..

[30] Katzmarzyk P.T., Friedenreich C., Shiroma E.J., Lee I.M. (2021). Physical Inactivity and Non-Communicable Disease Burden in Low-Income, Middle-Income and High-Income Countries. Br. J. Sports Med..

[31] Atella V., Piano Mortari A., Kopinska J., Belotti F., Lapi F., Cricelli C., Fontana L. (2019). Trends in Age-Related Disease Burden and Healthcare Utilization. Aging Cell.

[32] Davey A., Elias M.F., Siegler I.C. (2010). Cognitive Function, Physical Performance, Health, and Disease: Norms from the Georgia Centenarian Study. Exp. Aging Res..

[33] Teixeira L., Araújo L., Jopp D., Ribeiro O. (2017). Centenarians in Europe. Maturitas.

[34] Lepers R., Stapley P.J., Cattagni T. (2016). Centenarian Athletes: Examples of Ultimate Human Performance?. Age Ageing.

[35] Rosfjord H.S. (2021). New Metrics for Analysis and Presentation of Device-Based Indices of Physical Activity: Exploration of a Novel Way of Reducing Accelerometer Data into Analytical and Translational Indices of Physical Activity.

[36] Rowlands A.V., Edwardson C.L., Dawkins N.P., Maylor B., Metcalf K.M., Janz K.F. (2020). Physical Activity for Bone Health: How Much and/or How Hard?. Med. Sci. Sports Exerc..

[37] Rowlands A.V., Sherar L.B., Fairclough S.J., Yates T., Edwardson C.L., Harrington D.M., Davies M.J., Munir F., Khunti K., Stiles V.H. (2019). A Data-Driven, Meaningful, Easy to Interpret, Standardised Accelerometer Outcome Variable for Global Surveillance. J. Sci. Med. Sport.

[38] Arvidsson D., Fridolfsson J., Buck C., Ekblom Ö., Ekblom-Bak E., Lissner L., Hunsberger M., Börjesson M. (2019). Reexamination of Accelerometer Calibration with Energy Expenditure as Criterion: VO2net Instead of MET for Age-Equivalent Physical Activity Intensity. Sensors.

[39] Dawkins N.P., Yates T., Edwardson C.L., Maylor B., Davies M.J., Dunstan D., Highton P.J., Herring L.Y., Khunti K., Rowlands A.V. (2021). Comparing 24 h Physical Activity Profiles: Office Workers, Women with a History of Gestational Diabetes and People with Chronic Disease Condition(S). J. Sports Sci..

[40] Ekelund U., Tarp J., Steene-Johannessen J., Hansen B.H., Jefferis B., Fagerland M.W., Whincup P., Diaz K.M., Hooker S.P., Chernofsky A. (2019). Dose-Response Associations between Accelerometry Measured Physical Activity and Sedentary Time and All Cause Mortality: Systematic Review and Harmonised Meta-Analysis. BMJ.

[41] European Commission Ageing Europe (2020). Looking at the Lives of Older People in the EU (2020 Edition).

